# Chemical inhibitors of cyclin-dependent kinase (CDKi) improve pancreatic endocrine differentiation of iPS cells

**DOI:** 10.1007/s11626-023-00776-0

**Published:** 2023-07-05

**Authors:** Heming Ning, Ayumi Horikawa, Takayoshi Yamamoto, Tatsuo Michiue

**Affiliations:** 1 Department of Life Sciences (Biology), Graduate School of Arts and Sciences, The University of Tokyo, 3-8-1, Komaba, Meguro-Ku, Tokyo, 153-8902 Japan; 2 Department of Biological Sciences, Graduate School of Science, The University of Tokyo, 7-3-1 Hongo, Bunkyo-Ku, Tokyo, 113-0033 Japan

**Keywords:** iPS cells, Cyclin-dependent kinase, Pancreatic endocrine induction, Diabetes, Insulin

## Abstract

Islet transplantation, including pancreatic beta cells, has become an approved treatment for type I diabetes. To date, the number of donors limits the availability of treatment. Induction of pancreatic endocrine cells from pluripotent stem cells including iPSCs in vitro offers promise as a solution, but continues to face problems including high reagent costs and cumbersome differentiation procedures. In a previous study, we developed a low-cost, simplified differentiation method, but its efficiency for inducing pancreatic endocrine cells was not sufficient: induction of endocrine cells is non-uniform, resulting in colonies containing relatively high ratio of non-pancreatic-related cells. Here, we applied cyclin-dependent kinase inhibitors (CDKi) within a specific time window, which improved the efficiency of pancreatic endocrine cell induction. CDKi treatment reduced the prevalence of multi-layered regions and enhanced expression of the endocrine progenitor–related marker genes *PDX1* and *NGN3* resulting in enhanced production of both INSULIN and GLUCAGON. These findings support a step forward in the field of regenerative medicine of pancreatic endocrine cells.

**Supplementary Information:**

The online version contains supplementary material available at 10.1007/s11626-023-00776-0.

## Introduction

A well-known characteristic of type I diabetes mellitus is hyperglycemia resulting from the inability to produce insulin due to loss of beta cells. The current mainstream treatment for this disease is direct insulin injection. Several problems are associated with this treatment, including lifelong exogenous insulin dependence and unstable control of blood glucose levels (Gamble *et al*. 2018; Pathak *et al*. 2019). In recent years, transplantation of pancreatic islet cells has become a promising treatment to overcome the obstacles mentioned above. However, an insufficient number of donors and transplant rejection remain major challenges.

To overcome these challenges, the production of functional beta cells from induced pluripotent stem cells (iPS cells) for diabetes treatment has become a widely accepted challenge. Several bottlenecks exist in currently established methods for inducing beta cell differentiation from iPSCs. These bottlenecks include high cost due to numerous expensive reagents and lengthy procedures including cumbersome induction steps (D’Amour *et al*. 2006; Kroon *et al*. 2008; Kunisada *et al*. 2012; Pagliuca *et al*. 2014). To overcome these problems, in our previous study, we developed a new method (Horikawa *et al*. 2021) that utilizes fewer and less costly reagents and a simpler, shorter induction protocol. However, the differentiation level of the final product was found to be insufficient. For instance, in 2-dimensional culturing environment, differentiation seems not uniformly induced, resulting in scattered multiple layers of colonies containing non-pancreatic-related cells, recognized either as deep-colored areas through optical photomicrography or high DAPI signaling area through immunofluorescence imaging, which were defined as multi-layered regions. It has been reported that pancreatic endocrine cell genesis is enhanced by temporally regulated inhibition of cyclin-dependent kinase by treatment with CDK inhibitors (CDKi). CDK inhibition lengthens the cell cycle, preventing phosphorylation and subsequent degradation of NEUROG3 (NGN3), a key marker of pancreatic endocrine progenitors mainly utilizing human embryonic stem cells and mouse embryos (Krentz *et al*. 2017). We hypothesized that this treatment would reduce the prevalence of multi-layered regions and improve efficiency of endocrine differentiation. In this study, we examined the ability of CDKi to improve our protocol.

## Materials and methods

### Cell culture and differentiation

TkDN4-M cell line of human iPSCs (hiPSCs), which was established from human dermal fibroblasts cells, was used in this study (Takayama *et al*. 2010). hiPSCs were cultured on 6-well plates coated with Matrigel (Corning, Corning, NY) in serum-free mTeSR Plus medium (STEMCELL Technologies, Vancouver, Canada), without mouse embryonic fibroblast feeder cells, at 37 °C under 5% CO_2_ in air. hiPSCs were cultured with 10 µM Rho-associated kinase inhibitor (Y-27632; Adooq Bioscience, Irvine, CA) and passaged at a ratio of around 1:100–1:200 when hiPSCs reached about 80% confluency using Accutase (Gibco, Grand Island NY, Waltham, MA). Cells used for experiments had less than ten passages.

For definitive endoderm (DE) differentiation (stage 1), hiPSCs were dissociated with Accutase and plated at a density of about 2.5 × 10^4^ cells/cm^2^ on Matrigel-coated 4-well chamber slides (Thermo Fisher Scientific, Waltham, MA) with mTeSR Plus medium containing 10 μM Y-27632. After 1 d, the medium for the cultivation switched to DIF medium 1 (detailed below) with 3 µM CHIR99021 (Wako, Osaka, Japan), 100 ng/mL activin A (Ac), 0.25 mM vitamin C (Wako), and 10 μM Y-27632 for 24 h, and then cultured in DIF medium 1 with 100 ng/mL Ac, 0.25 mM vitamin C, and 10 μM Y-27632 for 48 h. In addition, undifferentiated iPS cells were cultured in mTeSR Plus medium for 3 d as negative control for endoderm-inducing efficiency check. At stage 2, cells were cultured for 6 d in DIF medium 2 (detailed below) with 1% B27 supplement (B27, Invitrogen, Waltham, MA), 10 μM SB431542 (SB, Wako), 0.5 μM LDN193189 (LDN, Sigma, St. Louis, MO), 2 μM retinoic acid (RA, Wako), and 0.25 mM vitamin C (VtC, Wako), and 10 μM Y-27632 was added at the first day of this stage. At stage 3, cells were cultured for 7 d in DIF medium 2 with 1% B27, 10 μM SB, 10 nM GLP-1 (Sigma), and 5 μM RepSox (Sigma). At stage 4, cells were cultured for 5 d in DIF medium 2 with 1% B27, 10 μM SB, and 10 nM GLP-1. Media were changed daily during stages 1 and 2, and every other day during stages 3 and 4.

Differentiation media used were as follows:

- DIF medium 1: modified DMEM medium (Cell Science & Technology Institute, Inc., Sendai, Japan) containing 10 µg/mL insulin (Wako), 5 µg/mL transferrin (Sigma), 500 µg/mL bovine serum albumin (BSA, Sigma), 10 µM sodium selenite, 10 µM ethanolamine (Sigma), and 10 µM 2-mercaptoethanol (Sigma).
- DIF medium 2: modified DMEM containing 5 µg/mL transferrin (Sigma), 500 µg/mL BSA, 10 µM sodium selenite, 10 µM ethanolamine, 10 µM 2-mercaptoethanol, and 7.5 ng/mL insulin-like growth factor (IGF-1, Sigma).

### Cyclin-dependent kinase inhibitor treatment

Treatment of CDK inhibitors (2.5 μM CDK4/6 inhibitor PD-0332991; Sigma), 1 μM CDK2 inhibitor II (EMD Millipore, Burlington, MA), and 1 μM CDK2 inhibitor III (EMD Millipore) was carried out. The inhibitors were added to the medium.

### Immunohistochemistry

Cells were fixed and immunostained with a standard protocol (Ninomiya *et al*. 2014). Antibodies used were listed as follows: rabbit anti-OCT4 antibody (1/500; Santa Cruz, Dallas, TX; sc-9081), goat anti-SOX17 antibody (1/300; R&D, Minneapolis, MN; AF1924), goat anti-PDX1 (1/300; R&D; AF2419), mouse anti-NGN3 (1/300; Developmental Studies Hybridoma Bank, Iowa City, IA; F25A1B3), mouse anti-GLUCAGON (1/600; Sigma-Aldrich, St. Louis, MO; G2654), rat anti-C-peptide (1/600; Developmental Studies Hybridoma Bank; GN-ID4), anti-rabbit IgG, Alexa Fluor 488 conjugated (1/600; Invitrogen; A21206), anti-goat IgG, Alexa Fluor 594 conjugated (1/600; Invitrogen; A11058), anti-mouse IgG, Alexa Fluor 488 conjugated (1/600; Invitrogen; A21202), anti-rat IgG, Alexa Fluor 594 conjugated (1/600; Invitrogen; A-11007). Nuclei were counterstained with DAPI (1/200; Dojindo, Kumamoto, Japan; 340–07971) before specimens were mounted in Prolong Gold Antifade Reagent (Invitrogen). Specimens were observed with an inverted fluorescent microscope (Keyence, Osaka, Japan).

### qRT-PCR

Total RNA was extracted using ISOGEN II (Nippongene, Tokyo, Japan). Reverse transcription was carried out using SuperScript III Reverse Transcriptase (Invitrogen). qRT-PCR was performed using KOD SYBR qPCR mix (TOYOBO, Osaka, Japan) or KAPA SYBR FAST One-Step qRT-PCR Kits (Sigma-Aldrich). Primer sets (Eurofins, Luxembourg City, Luxembourg) used for qRT-PCR are listed as follows:

**Table:** 

Gene	Forward sequence	Reverse sequence
*GAPDH*	GACATCAAGAAGGTGGTGAA	TGTCATACCAGGAAATGAGC
*OCT4*	CGAAAGAGAAAGCGAACCAGT	AACCACACTCGGACCACATCC
*NANOG*	CGCAAAAAAGGAAGACAAGGTCCC	GCATCCCTGGTGGTAGGAAGAGTAAG
*FOXA2*	TCTCCTCCATTGCTGTTGTTGC	ATTTCACCGTGTCAAGATTGGG
*SOX17*	CCTGGGTTTTTGTTGTTGCT	GAGGAAGCTGTTTTGGGACA
*PDX1*	TCCACCTTGGGACCTGTTTAGAG	CGAGTAAGAATGGCTTTATGGCAG
*NGN3*	CCCTCTACTCCCCAGTCTCC	CCTTACCCTTAGCACCCACA
*GLUCAGON*	CAGACCAAAATCACTGACAGGAAATA	ACATCCCACGTGGCTAGCA
*INSULIN*	AGCCTTTGTGAACCAACACC	GCTGGTAGAGGGAGCAGATG

Results shown are representative of at least three biological replicates

### Statistical analyses

Data are expressed as mean ± standard deviation. For comparisons of discrete data sets, paired *t*-test or Student’s *t*-test was used, as indicated in the legends. Two-tailed *p* < 0.05 was considered statistically significant.

## Results

**Figure 1.**
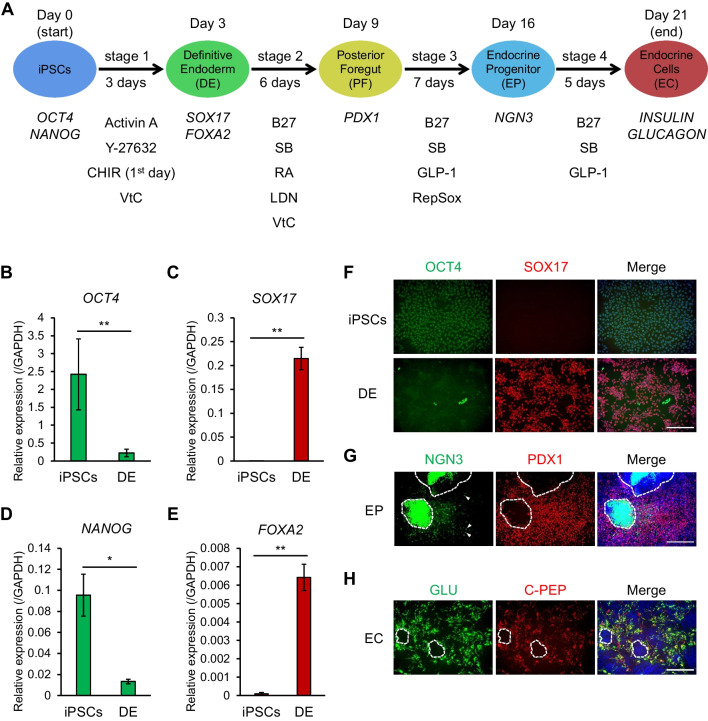
A protocol of pancreatic endocrine differentiating induction utilizing iPS cells. *A* Schematic illustration of pancreatic endocrine induction protocol. CHIR, CHIR99021; VtC, vitamin C; B27, supplement B27; SB, SB431542; RA, retinoic acid; LDN, LDN193189. *B*–*E* qRT-PCR analysis of gene expression of stemness markers, *OCT4* and *NANOG*, and endoderm markers, *SOX17* and *FOXA2*, normalized by *GAPDH* at stage 1. *Horizontal bars* indicate statistical analyses: *p < 0.05; **p < 0.01; ns, not significant; paired *t*-test. *F* IHC for OCT4 (*green*) and SOX17 (*red*) at stage 1. Nuclei were stained with DAPI (*blue*). *Scale bar*: 100 μm. *G* IHC for NGN3 (*green*) and PDX1 (*red*) at stage 3. *White arrowheads* indicate cells expressing NGN3. *White dashed box* indicates the multi-layered regions. Nuclei were stained with DAPI (*blue*). *Scale bar*: 100 μm. *H* IHC for GLUCAGON (GLU, *green*) and C-peptide (C-PEP, *red*) at stage 3. *White dashed box* indicates the multi-layered regions. Nuclei were stained with DAPI (*blue*). *Scale bar*: 100 μm.

We previously established a new protocol that differentiates iPS cells into INSULIN-producing cells with fewer steps and lower cost (Horikawa *et al*. 2021). This protocol, with slightly modified medium and reagents (see “Materials and methods” for detail), was applied in this research. Briefly, this protocol involves four stages of differentiation (Fig. 1*A*). At the end of stage 1, increased expression of the definitive endoderm markers *SOX17* and *FOXA2*, and reduced expression of the cell stemness markers *OCT4* and *NANOG* were observed by qRT-PCR (Fig. 1*B*–*E*). This trend was confirmed by immunohistochemistry (IHC) (Fig. 1*F*). At stage 3, where the posterior foregut differentiates into endocrine progenitor, cells positive for PDX1 and NGN3 were detected. Although expression of both NGN3 and PDX1 was detected in the mono-layered regions, in which DAPI intensity was relatively low, PDX1 was rarely detected in multi-layered regions, where DAPI intensity was higher (Fig. 1*G*). This suggests that the cells in the multi-layered region are differentiating into non-endocrine cells because co-expression of both NGN3 and PDX1 is essential for endocrine cell identity (Weng *et al*. 2020). At the final stage of differentiation (stage 4), a certain number of cells expressing C-peptide and GLUCAGON were consistently detected in mono-layered regions, whereas in multi-layered regions, fewer C-peptide- and GLUCAGON-positive cells were found (Fig. 1*H*). Thus, the formation of multi-layered regions seems to correlate with low efficiency differentiation to endocrine cells.

**Figure 2.**
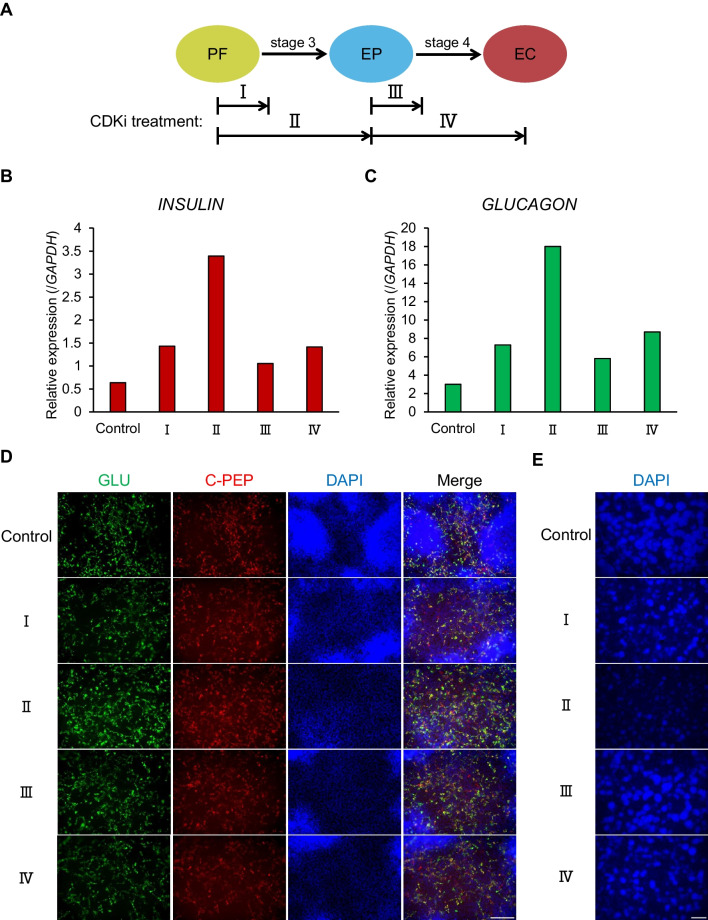
Time window determination for CDKi treatment. *A* Time window of CDKi treatment. *Roman numerals* indicate different periods of treatment: treatment at the first day of stage 3 (I), throughout stage 3 (II), at the first day of stage 4 (II), or throughout stage 4 (IV). *B*, *C* RT-PCR analysis of expression of *INSULIN* (*B*) and *GLUCAGON* (*C*). *D* IHC for GLUCAGON (GLU, *green*) and C-peptide (C-PEP, *red*) with DAPI (*blue*). *Scale bar*: 100 μm. *E* IHC for DAPI (*blue*) at zoom rate of 20 × . *Scale bar*: 500 μm.

To further investigate the development of multi-layered regions, we observed cells over time, and it revealed that cells tend to form multi-layered regions over time (Fig. 2*B*), especially during stages of endocrine fate determination (days 9–16). In a previous report, combined treatment with CDK4/6 and CDK2 inhibitors (CDK inhibitors, CDKi) enhanced endocrine cell fate determination by stabilizing the NGN3 protein (Krentz *et al*. 2017). Because the reported time range for this treatment (Krentz *et al*. 2017) was similar to the period of multi-layered region formation in our induction system (although these researchers mainly used different cell types, embryonic stem cells, or mouse embryos), we predicted that treatment would reduce multi-layered regions by limiting cell proliferation. Although our protocol yielded relatively low NGN3 expression (Fig. 1*G*), this was also expected to be elevated by CDKi treatment. We thus incorporated CDK4/6 and CDK2 inhibitors into our protocol.

**Figure 3.**
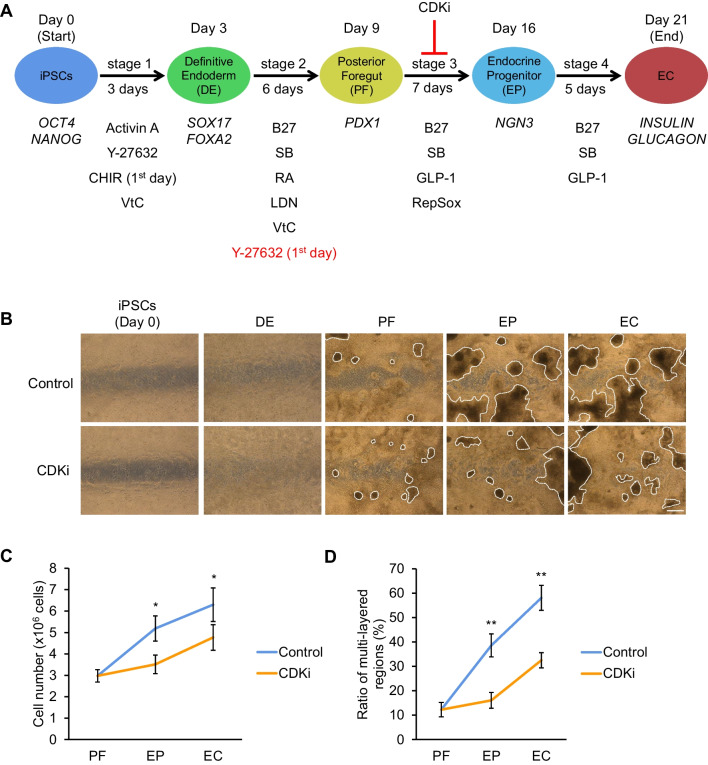
CDKi treatment reduced the multi-layered region. *A* Schematic illustration of protocol of pancreatic endocrine differentiation with CDK inhibitor. *B* Bright field image of differentiating cells. *Dashed box* indicates the multi-layered region. *Scale bar*: 100 μm. *C* Cell number at the end of stages 2 (PF, day 11), 3 (EP, day 18), and 4 (EC, day 23). *D* Ratio of the multi-layered region at days 18 and 23, corresponding to *B*. *n* = 3, including three biological and one technical replicates.

To determine the optimal time window for CDKi treatment in our protocol, four different time points (Fig. 2*A*) for CDKi addition were examined. qRT-PCR analysis revealed that samples treated with CDKi throughout stage 3 (days 9–15) displayed upregulated *INSULIN* and *GLUCAGON* expression compared with the control group and cells treated during other periods (Fig. 2*B*, *C*), although this experiment was performed only once. This tendency was confirmed by IHC analysis on various zoom rates with apparent difference in the size of high DAPI areas (Fig. 2*D*, *E*). Based on these results, CDKi treatment throughout stage 3 was used in subsequent experiments (Fig. 3*A*). It should be noted that, after selecting this time window for CDKi treatment, we faced the problem of cells peeling off on the first day of stage 2. When we applied Y-27632 for an additional 24 h, until the end of the first day of stage 2, cell attachment improved. This modification was applied in the following experiment (Fig. 3*A*). With this new protocol, we first confirmed the inhibitory effect of CDKi on proliferation rate: the total number of cells decreased in the CDKi group (Fig. 3*C*), and the ratio of multi-layered regions was significantly reduced (Fig. 3*B*, *D*) in three repetitive experiments.

**Figure 4.**
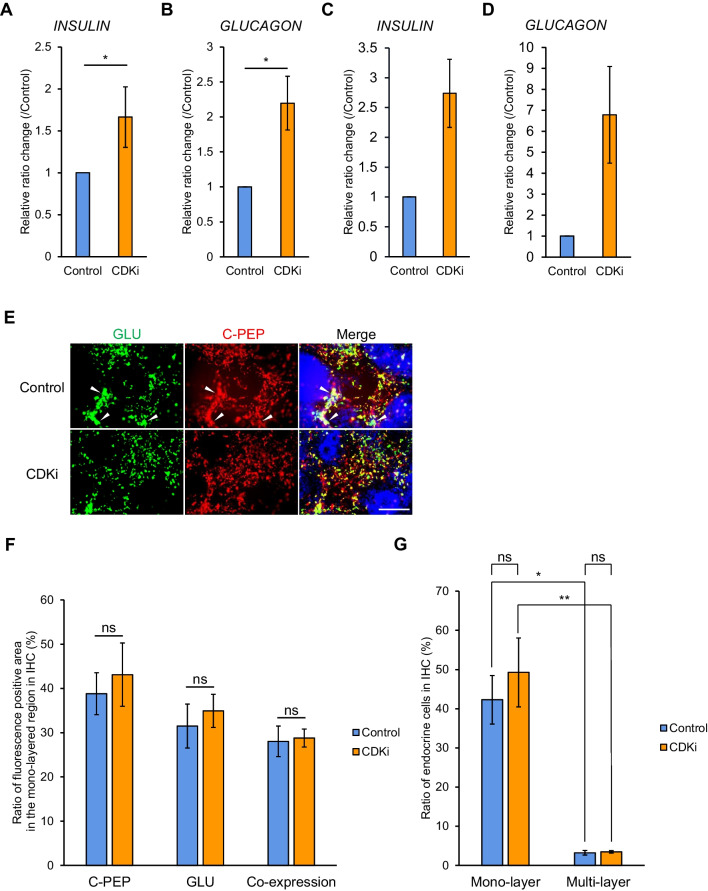
CDKi treatment improved pancreatic endocrine induction in iPSCs. *A*, *B* qRT-PCR analysis of expression of *INSULIN* (*A*) and *GLUCAGON* (*B*), normalized by the control group at stage 4 (*n* = 6). *Horizontal bars* indicate statistical analyses: *p < 0.05; ns, not significant; paired *t*-test. *C*, *D* qRT-PCR analysis of expression of *INSULIN* (*C*) and *GLUCAGON* (*D*) without Y27362 addition at day 5 of induction, normalized by the control group at stage 4, *n* = 6. *Horizontal bars* indicate statistical analyses: *p < 0.05; paired *t*-test. *E* IHC for GLUCAGON (GLU, *green*) and C-peptide (C-PEP, *red*) at stage 4. *Arrowhead* indicates the area between mono- and multi-layered regions with high intensity of GLU/C-PEP. Nuclei were stained with DAPI (*blue*). *Scale bar*: 100 μm. *F* Area percentage of cells expressing C-peptide and/or GLUCAGON, which are defined as the endocrine cells, in the mono-layered region, corresponding to *C* (*n* = 15, including three biological and five technical replicates). ns, not significant; Student’s *t*-test. *G* Ratio of endocrine cells, which express INSULIN and/or GLUCAGON in mono-layered or multi-layered region, corresponding to *C* (*n* = 15, including three biological and five technical replicates). ns, not significant; Student’s *t*-test.

Although culture conditions were altered by extending application of Y-27632 by 24 h, qRT-PCR results of three repeated experiments showed that expression levels of *INSULIN* and *GLUCAGON* were significantly increased by nearly 2-fold (Fig. 4*A*, *B*), as in the previous protocol (Fig. 4*C*, *D*). However, in mono-layered regions, no significant change in the ratio of the area of endocrine cells (C-peptide- and/or GLUCAGON-positive cells) due to CDKi treatment was detected (Fig. 4*E*, *F*). We wondered whether the endocrine cell ratio was increased in the multi-layered regions, but not in the mono-layered regions. However, this was not the case: the ratio of endocrine cells to all cells showed no significant difference with or without CDKi in the multi-layered regions (Fig. 4*G*). However, the ratio of endocrine cells was higher in the mono-layered regions than in the multi-layered regions (Fig. 4*G*). Thus, we hypothesized that the CDKi treatment–induced expansion of mono-layered region would explain the upregulation of *INSULIN* and *GLUCAGON* expression levels detected by qRT-PCR. To examine this, we re-examined the samples with less-magnified images and found that the multi-layered regions, the ratio of which was decreased by CDKi treatment, contained just little *INSULIN*- or *GLUCAGON*-expressing cells at the final stage (Figure S1*A*, *B*).

**Figure 5.**
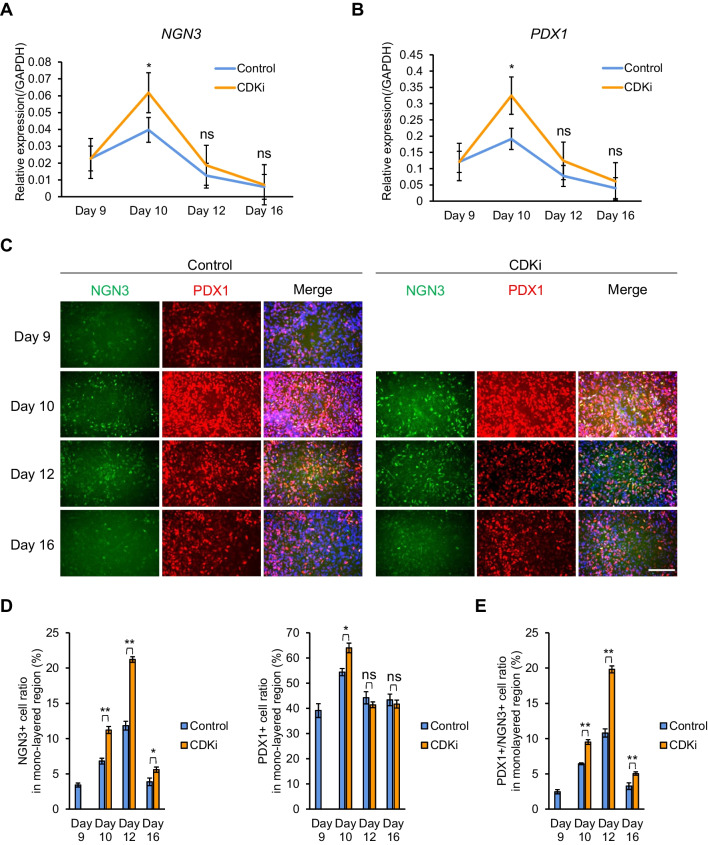
CDKi treatment enhanced NGN3 and PDX1 expression in the mono-layered region. *A*, *B* qRT-PCR analysis of expression of *NGN3* (*A*) and *PDX1* (*B*) (*n* = 9, including three biological and three technical replicates), normalized by *GAPDH*. Expression of both markers showed an overall increase in the CDKi-treated group. *C* Comparison of IHC for NGN3 (*green*) and PDX1 (*red*) between the control and CDKi-treated group. Nuclei were stained with DAPI (*blue*). *Scale bar*: 100 μm. *D* Area percentage of cells expressing PDX1 (*left*) and NGN3 (*right*) in 1.34 mm^2^ foci from the mono-layered region of IHC photograph in *C*. *p < 0.05, **p < 0.01, ***p < 0.001; Student’s *t*-test. *E* Area percentage of cells co-expressing PDX1 and NGN3 in the mono-layered regions, corresponding to *D*. *p < 0.05, **p < 0.01; Student’s *t*-test.

To acquire deeper insight into CDKi treatment during stage 3, we conducted time-course analyses of CDKi-treated cells. Expression of both *PDX1* and *NGN3* peaked 24 h after entering stage 3 in control and CDKi groups, and then slowly dropped to starting levels throughout the remainder of stage 3 (Fig. 5*A*, *B*). Peak levels of both *PDX1* and *NGN3* in the CDKi-treated group were higher than those in the control group (Fig. 5*A*, *B*). In agreement with qRT-PCR results, IHC showed that the ratio of NGN3 positive cells increased, and then gradually decreased after reaching its peak in the mono-layered region, as did the fraction of PDX1/NGN3 co-expressing cells in the mono-layered regions. In addition, IHC analysis confirmed that the fraction of NGN3^+^ cells in mono-layered regions was significantly increased by CDKi treatment throughout stage 3, suggesting increased penetrance in endocrine cell fate determination in iPSCs due to CDKi treatment. Although the *PDX1* mRNA level was higher in the CDKi group than in the control group, the area size of PDX1^+^ cells was slightly lower in the CDKi group than in the control group (Fig. 5*D*). These differences may be explained by our methods. RNA expression of *PDX1* was measured over the whole region by qRT-PCR (including mono- and multi-layered regions), while the area covered by PDX1^+^ cells was measured only in mono-layered regions, which were increased by CDKi. Taken together, our results suggest that CDKi treatment increases pancreatic endocrine progenitor cells by reducing the total area of multi-layered regions and increasing the number of NGN3/PDX1 co-expressing cells in mono-layered regions.

## Discussion

Cyclin-CDK complexes, such as CDK1/CYCLIN B (M phase), CDK2/CYCLIN A (S phase), CDK2/CYCLIN E1 (late G1 phase), and CDK4/CYCLIN D1 (G1 phase), phosphorylate discrete sites on NGN3 (Azzarelli *et al*. 2017). Phosphorylation decreases NGN3 stability and DNA binding capability, interfering with expression of its target genes (Krentz *et al*. 2017), suggesting that CDK activity regulates NGN3-mediated differentiation. Indeed, lengthening G1/S phase by restricting CDK2 and CDK4/6 activity decreases NGN3 phosphorylation, retards cell cycle progression, and enhances endocrine cell fate decisions (Krentz *et al*. 2017). However, NGN3 induces cyclin-dependent kinase inhibitor 1a (cdkn1a) expression, thereby inhibiting cell proliferation. This seems to be a discrepancy: cell cycle inhibition induces NGN3, but NGN3 inhibits cell proliferation. However, downregulation of NGN3 at a certain time point after cells are specified as endocrine progenitors allows expansion of the mature islet cell population (Miyatsuka *et al*. 2011). Moreover, CDK4, a G1/S-specific CDK, is highly expressed in pancreatic epithelium, increasing the number of endocrine progenitors (Kim and Rane 2011). These results further suggest the importance of limiting NGN3 levels after determining endocrine progenitor fate. Similarly, we removed CDKi after endocrine progenitor induction and succeed in enhancing endocrine cell differentiation. On the other hand, due to the inhibitory effect of CDKi upon proliferation of cell, the expansion of PDX1/NGN3 co-expressing endocrine progenitor cells might have been limited as well. Thus, it is thought that, although PDX1/NGN3 co-expressing cells significantly increased at early phase of stage 3, the change of the ratio of endocrine cells in the mono-layered region by CDKi treatment was insufficient at stage 4. Given these results, it is important to determine the appropriate stage/time window to inhibit cell cycle progression. In addition, several commonly recognized exogenous physical approaches have been reported interfering cell cycle, including temperature (Falahati *et al*. 2021), hypoxia (Druker *et al*. 2021), and mechanical interactions (Gupta and Chaudhuri 2022). Considering the importance of cell cycle regulation in beta cell differentiation, these methods might provide new perspective for enhancing pancreatic endocrine induction as well.

The multi-layered regions are considered to be composed of non-endocrine-related cells because IHC demonstrated low presence of C-peptide/GLU-positive cells (Figure S1*A*). As mentioned in the “Introduction,” NGN3 level was relatively higher in the multi-layered regions comparing with surrounding mono-layered regions as IHC showed. On the other hand, during the earlier stage of induction, we noticed that small amount of iPS cells did not successfully differentiate into endocrine cells; it is possible that these proportions of cells continued to differentiate into other types of cells under the influence of the reagents we added during induction, including SB431542, LDN193189, and retinoic acid. Considering the role these reagents played in other directed paths of differentiation and the phenotype of NGN3 in our research, there is possibility that the main component of multi-layered regions could be neuron-related cells. However, relatively high densities of C-peptide-positive cells were present in the margins of the multi-layered regions (Fig. 4*E*). Recent research has demonstrated the positive influence of co-cultivating multiple cell types on differentiation. For instance, osteogenic differentiation of mesenchymal stem cells can be stimulated by co-culture with several different types of cells, including osteocytes and osteoblasts, in a simplified bone niche (Birmingham *et al*. 2012). Neural differentiation of adipose tissue–derived stem cells can also be improved by co-culture with embryonic stem cells (Bahmani *et al*. 2014). Taken together, these cited studies and our results suggest that multi-layered regions may exert a positive influence on endocrine differentiation.

## Conclusion

In summary, we examined the effects of cyclin-dependent kinase inhibitors (CDKi) on induction of pancreatic endocrine cells and found that this treatment enhances expression of the endocrine progenitor–related genes *NGN3* and *PDX1*, while decreasing population of non-pancreatic-related cells by inhibiting cell proliferation, resulting in enhanced endocrine differentiation.

## Supplementary Information

Below is the link to the electronic supplementary material.

Supplementary file1 CDKi reduced overall ratio of multi-layered region. (A) IHC for GLUCAGON (GLU, green) and C-peptide (C-PEP, red) at stage 4 with zoom rate of 20x. Nuclei were stained with DAPI (blue). Scale bar: 500 μm. (B) Ratio of the multi-layered region (high DAPI region), measured by the result in F. ***p < 0.001; Student’s t-test. (PPTX 3095 KB)

## Data Availability

All relevant data are within the manuscript and its Supporting Information files.
