# Supplementary material for: Chemical inhibitors of cyclin-dependent kinase (CDKi) improve pancreatic endocrine differentiation of iPS cells
Source: In Vitro Cell Dev Biol Anim. 2023 Jul 5;59(6):410–9. doi: 10.1007/s11626-023-00776-0 (PMC10374832; doi:10.1007/s11626-023-00776-0)
Supplement: Supplementary file 1 — Supplementary file1 CDKi reduced overall ratio of multi-layered region. (A) IHC for GLUCAGON (GLU, green) and C-peptide (C-PEP, red) at stage 4 with zoom rate of 20x. Nuclei were stained with DAPI (blue). Scale bar: 500 μm. (B) Ratio of the multi-layered region (high DAPI region), measured by the result in F. ***p < 0.001; Student’s t-test. (PPTX 3095 KB) [file 11626_2023_776_MOESM1_ESM.pptx]

## Slide 1
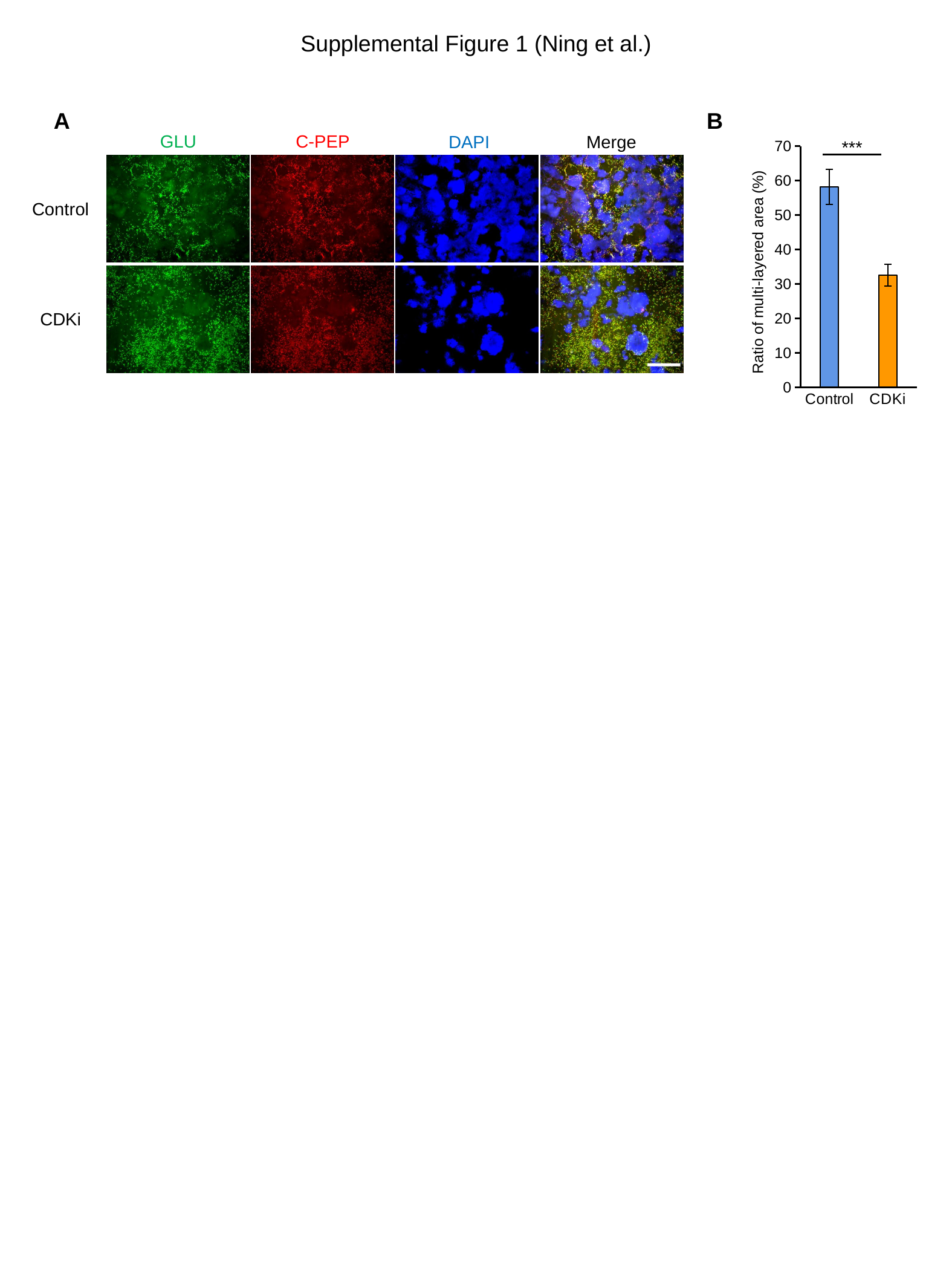

Supplemental Figure 1 (Ning et al.)
A
B
GLU
C-PEP
DAPI
Merge
***
### Chart
| Category | |
|---|---|
| Control | 58.1156666666667 |
| CDKi | 32.533 |
Control
CDKi

## Slide 2
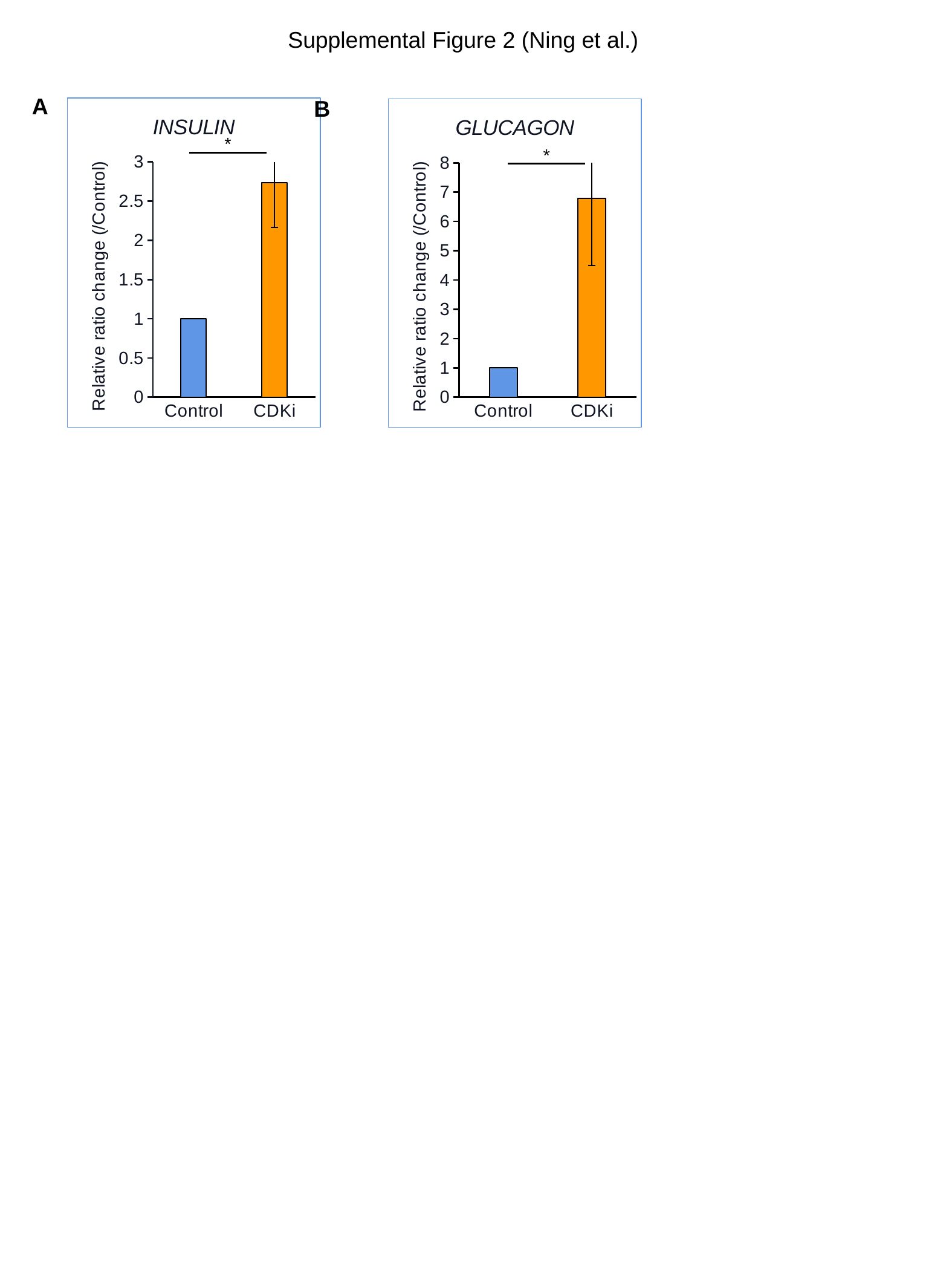

Supplemental Figure 2 (Ning et al.)
A
B
### Chart: INSULIN
| Category | |
|---|---|
| Control | 1.0 |
| CDKi | 2.73802028291607 |
### Chart: GLUCAGON
| Category | |
|---|---|
| Control | 1.0 |
| CDKi | 6.78979314554832 |*
*
